# The highly pathogenic H5N1 avian influenza virus induces the mitogen-activated protein kinase signaling pathway in the trachea of two Ri chicken lines

**DOI:** 10.5713/ab.21.0420

**Published:** 2022-01-05

**Authors:** Thi Hao Vu, Yeojin Hong, Anh Duc Truong, Sooyeon Lee, Jubi Heo, Hyun S. Lillehoj, Yeong Ho Hong

**Affiliations:** 1 Department of Animal Science and Technology, Chung-Ang University, Anseong 17546, Korea; 2 Department of Biochemistry and Immunology, National Institute of Veterinary Research, 86 Truong Chinh, Dong Da, Hanoi 100000, Vietnam; 3 Animal Biosciences and Biotechnology Laboratory, Agricultural Research Services, United States Department of Agriculture, Beltsville, MD 20705, USA

**Keywords:** H5N1, RNA Sequencing, Mitogen-activated Protein Kinase (MAPK) Signaling Pathway, Ri Chicken

## Abstract

**Objective:**

The highly pathogenic avian influenza virus (HPAIV) is a threat to the poultry industry and economy and remains a potential source of pandemic infection in humans. Antiviral genes are considered a potential factor for studies on HPAIV resistance. Therefore, in this study, we investigated gene expression related to the mitogen-activated protein kinase (MAPK) signaling pathway by comparing non-infected, HPAI-infected resistant, and susceptible Ri chicken lines.

**Methods:**

Resistant (*Mx*/A; *BF2*/B21) and susceptible Ri chickens (*Mx*/G; *BF2*/B13) were selected by genotyping the *Mx* and *BF2* genes. Then, the tracheal tissues of non-infected and HPAIV H5N1 infected chickens were collected for RNA sequencing.

**Results:**

A gene set overlapping test between the analyzed differentially expressed genes (DEGs) and functionally categorized genes was performed, including biological processes of the gene ontology (GO) and Kyoto encyclopedia of genes and genomes (KEGG) pathways. A total of 1,794 DEGs were observed between control and H5N1-infected resistant Ri chickens, 432 DEGs between control and infected susceptible Ri chickens, and 1,202 DEGs between infected susceptible and infected resistant Ri chickens. The expression levels of MAPK signaling pathway-related genes (including *MyD88*, *NF-κB*, *AP-1*, *c-fos*, *Jun*, *JunD*, *MAX*, *c-Myc*), cytokines (IL-1β, IL-6, IL-8), type I interferons (IFN-α, IFN-β), and IFN-stimulated genes (*Mx1*, *CCL19*, *OASL*, and *PRK*) were higher in H5N1-infected than in non-infected resistant Ri chickens. MyD88, Jun, JunD, MAX, cytokines, chemokines, IFNs, and IFN-stimulated expressed genes were higher in resistant-infected than in susceptible-infected Ri chickens.

**Conclusion:**

Resistant Ri chickens showed higher antiviral activity compared to susceptible Ri chickens, and H5N1-infected resistant Ri chickens had immune responses and antiviral activity (cytokines, chemokines, interferons, and IFN-stimulated genes), which may have been induced through the MAPK signaling pathway in response to H5N1 infection.

## INTRODUCTION

Avian influenza virus (AIV) belongs to the genus *Influenzavirus* A in the *Orthomyxoviridae* family [1]. AIV is classified as either highly pathogenic avian influenza (HPAI) or low pathogenic avian influenza (LPAI) based on genetics and disease severity. The HPAI virus can kill up to 90% to 100% of flocks and the disease can spread rapidly, devastating the poultry industry [2]. Moreover, H5N1, a type of HPAI virus, is a threat to the poultry industry as well as the economy and remains a potential source of pandemic infection in humans [3].

Mx proteins, members of the dynamin family of large GTPases, inhibit the activity or trafficking of viral polymerase to prevent viral RNA replication [4]. Several previous reports have shown that only the asparagine (Asn-AAT) polymorphism at the 631st position triggers antiviral activity, whereas Mx proteins carrying a serine (Ser-AGT) at that position and do not suppress viral growth [5]. In addition, the major histocompatibility complex haplotype can also affect the antiviral activity of the host [6,7]. Previous research has shown a significant association between the *BF2*/B21 haplotype and resistance to several pathogens, including infectious bursal disease virus [6], and AIV [7], whereas the *BF2*-B13 haplotype is not. Furthermore, our previous study showed that resistant Ri chicken lines had higher antiviral activity than susceptible Ri chickens [8].

The mitogen-activated protein kinase (MAPK) signaling pathway and protein-serine/threonine kinases control several cellular activities, such as activation, proliferation, differentiation, and apoptosis [9]. H5N1 AIV can activate key host signaling pathways, including the MAPK signaling pathway [10]. Furthermore, the expression of type I interferons and IFN-stimulated genes can be regulated by the MAPK signaling pathway genes (*JNK* and *p38*) through the activating protein-1 (AP-1) transcription factor or the phosphorylation of Tyr701 and Ser727 in STAT1 in response to influenza A virus infections [11,12]. Interestingly, many miRNAs that are related to MAPK signaling pathway molecules are differentially expressed in the exosomal miRNA of non-infected and H5N1-infected resistant Ri chickens [13].

In this study, we used resistant and susceptible Ri chicken lines, a local chicken breed in Vietnam, as an experimental animal [8]. Chickens resistant and susceptible to HPAIV were differentiated by genotyping their *Mx*(A/G) and *BF2*(B21/B13) genes. These chickens were infected with HPAIV H5N1 and gene expression patterns in the trachea tissue were analyzed using high-throughput RNA sequencing. We analyzed the expression of genes related to the MAPK signaling pathway in control, resistant, and susceptible H5N1-infected chickens.

## MATERIALS AND METHODS

### Experimental chickens and HPAIV infection

All 4-week-old Ri chickens (5 chickens/group, Supplementary data; Supplementary Table S1) were kept in specific-pathogen-free conditions and observed daily for signs of disease and death. All experiments, including chicken management, HPAIV infection, and sample collection, were conducted in our collaborative lab at the Department of Biochemistry and Immunology at the National Institute of Veterinary Research (NIVR), Vietnam.

For the *Mx* gene, Ri chickens that have a non-synonymous adenine single nucleotide polymorphism at residue 631 were genotyped as resistant. In addition, for the *BF2* gene, those with the B21 genotype were found to be resistant. Taken together, HPAIV-resistant Ri chickens had the genotype *Mx*(A)/*BF2*(B21) [13]. To clarify the gene expression after H5N1 infection between two chicken lines, we conducted with three comparison groups: susceptible control vs infection, resistant control vs infection, and resistant infection vs. susceptible infection in the trachea samples after three days of H5N1 infection. In the resistant and susceptible control groups, five chickens per group were inoculated intranasally with phosphate-buffered saline. In the resistant and susceptible H5N1 infection group, five Ri chickens per group were inoculated intranasally with the collected allantoic fluid, including 10^4^ egg infectious doses (EID50) of A/duck/Vietnam/QB1207/2012 (H5N1), based on the Office International des Epizooties (OIE) guidelines [14]. The care and experimental use of the chickens were approved by the Ministry of Agriculture and Rural Development of Vietnam (TCVN 8402:2010/TCVN 8400-26:2014).

### Sample collection and total RNA preparation

At three days post-infection, tracheal tissues were collected from five chickens per group, according to the World Health Organization Manual on Animal Influenza Diagnosis and Surveillance. Total RNA from tracheal tissue was extracted using TRIzol reagent (Invitrogen, Carlsbad, CA, USA), according to the manufacturer’s instructions.

### High-throughput RNA sequencing and data analysis

The RNA sequencing was conducted by LAS (Gyeonggi, Korea) using an Illumina MGISEQ platform (Illumina Inc., San Diego, CA, USA). The raw sequence reads were filtered based on quality using FastQC version 0.11.5 (http://www.bioinformatics.babraham.ac.uk/projects/fastqc/). Potentially existing sequencing adapters and low-quality bases in the raw reads were trimmed using Skewer version 0.2.2 [15]. The cleaned high-quality reads after trimming the low-quality bases and sequencing adapters were mapped to the reference genome using STAR software version 2.5 [16]. Sequencing libraries were prepared in a strand-specific manner using the Illumina strand-specific library preparation kit MGIEasyRNA Directional Library Prep Kit (Illumina Way, San Diego, CA, USA). The gene annotation of the reference genome, gg6, from the UCSC chicken genome (https://genome.ucsc.edu) was used as a gene model, and the expression values were calculated in fragments per kilobase of transcript per million fragments mapped (FPKM) units. The differentially expressed genes between the two selected biological conditions were analyzed using Cuffdiff software version 2.2.1 in the Cufflinks package [17]. The scatter plots for the gene expression values and the volcano plots for the expression-fold changes and p-values between the two selected samples were also drawn using in-house R scripts. The assignment to significance was determined using a threshold. The default threshold was |fold-change|≥2 and p-value<0.05. To elucidate the biological functional role of the differential gene expression between the compared biological conditions, a gene set overlapping test between the analyzed differentially expressed genes and functionally categorized genes, including the biological processes of gene ontology (GO), Kyoto encyclopedia of genes and genomes (KEGG) pathways, and other functional gene sets was done using g:Profiler version 0.6.7 (https://biit.cs.ut.ee/gprofiler/gost).

### MAPK signaling pathway enrichment analysis

All transcript coding genes were aligned with the Search and Color Pathway tool of KEGG Mapper (https://www.genome.jp/kegg/tool/map_pathway2.html) to identify the upregulated or downregulated DEGs in the three comparisons. The heatmaps were generated using MeV4.9 software with |log2| fold change values between HPAI compared to the control, or the HPAI-resistant group compared to the HPAI-susceptible group. The protein–protein interactions of the MAPK signaling pathway-related genes were analyzed using the STRING program (version 11.0; http://string-db.org/).

### Quantitative real-time polymerase chain reaction validation for MAPK signaling pathway genes

Quantitative real-time polymerase chain reaction (qRT-PCR) was performed to verify the DEGs obtained from RNA sequencing. Before cDNA synthesis, 2 μg of total RNA was treated with 1.0 U DNase I (Thermo Fisher Scientific, Waltham, MA, USA) to remove potentially contaminating genomic DNA. The cDNA synthesis was performed using a RevertAid First Strand cDNA Synthesis Kit (Thermo Fisher Scientific, USA), according to the manufacturer’s instructions. The primer sequences of 13 genes and the housekeeping gene, glyceraldehyde-3-phosphate dehydrogenase (*GAPDH*) were designed using Primer-BLAST (http://www.ncbi.nlm.nih.gov/tools/primer-blast/) (Table 1). The qRT-PCR was performed in a LightCycler 96 system (Roche, Indianapolis, IN, USA) using the Dyne qPCR 2X PreMIX (Dyne Bio, Seongnam, Korea), according to the manufacturer’s recommendations. Relative gene expression was calculated using the 2^−^^ΔΔ^^Ct^ method after normalization with chicken *GAPDH* [18]. All experiments were performed independently in triplicates.

### Statistical analysis

Statistical analysis was carried out using SPSS software (IBM, SPSS 26.0 for Windows, Chicago, IL, USA), and p<0.05 was considered statistically significant in differences between treatment means generated using Student’s *t*-test. All qRT-PCR experiments were replicated independently three times, and the mean±standard error of the mean values for each group were reported.

## RESULTS

### Analysis of RNA sequencing data

After H5N1 AIV infection, we observed that the chickens had ruffled hair and tracheal hemorrhage. Among the tracheal tissue samples obtained from control and H5N1-infected Ri chickens three days post-H5N1 infection, only the quality control check-passed samples were subjected to high-throughput RNA sequencing: resistant control group (R1D3C, R2D3C, and R4D3C); resistant infection (R1D3I, R2D3I, R3D3I, and R5D3I), susceptible control (S2D3C and S3D3C), and susceptible infection (S1D3I, S2D3I, S3D3I, and S5D3I) (Supplementary Table S2).

Supplementary Table S2 shows the statistics of the raw and clean reads of individual sample transcriptomes after sequence processing and analysis. The 11 libraries produced between 5.3 GB and 9.4 GB worth of cDNA sequences per chicken. After data filtering, between 17.9 million and 31.6 million clean reads were obtained for each sample from the non-infected and H5N1-infected chicken lines.

The number of mapped reads, percentages, and transcripts are shown in Supplementary Table S3. More than 84.3% of the filtered reads from each library were mapped to the reference genome.

### Identification of differentially expressed genes and data analysis

**Figure 1:**
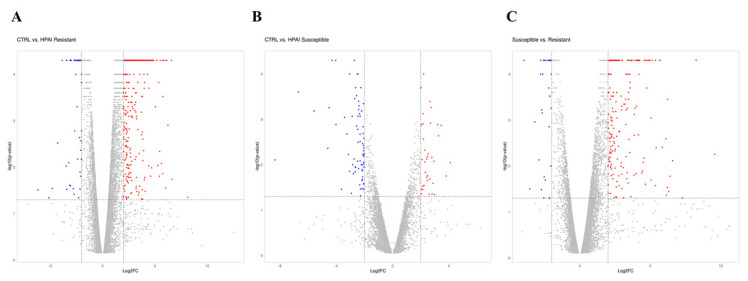
Distribution of DEGs represented in a Volcano plot. (A) D3-CTRL vs D3-HPAI infected resistant, (B) D3-CTRL vs D3-HPAI infected susceptible chickens, (C) D3-HPAI infected resistant vs D3-HPAI infected susceptible chickens for |log_2_| (fold-change) and –log_10_ (p-value) to show expression change and its significance. The blue dots indicate DEGs that have significantly downregulated; red dots indicate DEGs that have significantly upregulated; the gray dots indicate no significant differences between the two groups. DEGs, differentially expressed genes; HPAI, highly pathogenic avian influenza.

A gene was identified as significantly changed if the absolute value of the fold change was greater than 2 (up or down) and the p-value was less than 0.05 in comparison to the control group (Figure 1A–C). As shown in the volcano plot, of the 1,794 DEGs, 1,336 genes (74%) were significantly upregulated, and 458 genes (26%) were downregulated after HPAIV-H5N1 infection in the resistant Ri chicken line (Figure 1A). Among the 432 DEGs, 189 genes (44%) were significantly upregulated, and 243 genes (56%) were downregulated after HPAIV-H5N1 infection in the susceptible Ri chicken line (Figure 1B). A total of 1,202 DEGs were screened, with 854 genes (71%) significantly upregulated, and 348 genes (29%) downregulated in the HPAI-resistant group compared to the HPAI-susceptible group (Figure 1C).

**Figure 2:**
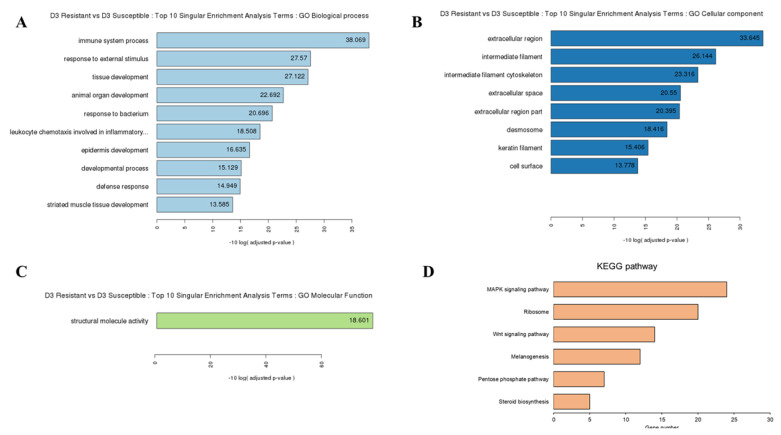
Gene ontology (GO) functional analysis for HPAI-infected resistant and HPAI-infected susceptible Ri chicken lines. The enriched biological terms include: (A) Top 10 singular enrichment analysis terms (SEA): GO biological process, (B) GO cellular component, and (C) GO molecular function (D) Kyoto encyclopedia of genes and genomes functional pathways from the 1,202 DEGs in HPAI infected resistant and HPAI infected susceptible Ri chicken lines obtained by criteria (|fold-change|≥2)∩ (p<0.05). GO, gene ontology; DEGs, differentially expressed genes; HPAI, highly pathogenic avian influenza.

To explore the function of these DEGs, we performed GO and KEGG pathway analyses. We identified 74, 10, and 11 subcategories of the biological process, cellular component, and molecular function in resistant chicken after HPAI infection, respectively (Supplementary Figure S1A-C; Supplementary Table S4). The KEGG pathway analysis revealed ten categories of immune-related pathways, including influenza A (14 DEGs), cytokine-cytokine receptor interaction (11 DEGs), herpes simplex infection (11 DEGs), Toll-like receptor signaling pathway (11 DEGs), p53 signaling pathway (10 DEGs), nucleotide-binding oligomerization domain-like receptor (NOD-like receptor) signaling pathway (9 DEGs), RIG-I-like receptor signaling pathway (7 DEGs), *Salmonella* infection (5 DEGs), cytosolic DNA-sensing pathway (5 DEGs), and the AGE-RAGE signaling pathway in diabetic complications (3 DEGs) (Supplementary Figure S1D; Supplementary Table S4). We identified 22, 15, and 6 subcategories of the biological process, cellular component, and molecular function in susceptible chicken after HPAI infection, respectively (Supplementary Figure S2A-C; Supplementary Table S5). The KEGG pathway analysis revealed three categories of immune-related pathways, including ribosomes (20 DEGs), tight junctions (10 DEGs), and cardiac muscle contraction (8 DEGs) (Supplementary Figure S2D; Supplementary Table S5). We identified 13, 8, and 1 subcategories of the biological process, cellular component, and molecular function in the HPAI-resistant compared to the HPAI-susceptible group, respectively (Supplementary Figure 2A-C; Supplementary Table S6). The KEGG pathway analysis revealed six categories of immune-related pathways, including the MAPK signaling pathway (24 DEGs), ribosome (20 DEGs), Wnt signaling pathway (14 DEGs), melanogenesis (12 DEGs), pentose phosphate pathway (7 DEGs), and steroid biosynthesis (5 DEGs) (Figure 2D; Supplementary Table S6). Most of the DEGs in the HPAI-resistant and HPAI-susceptible comparisons were related to the MAPK signaling pathway.

### Analysis of MAPK signaling pathway with DEGs

**Figure 3:**
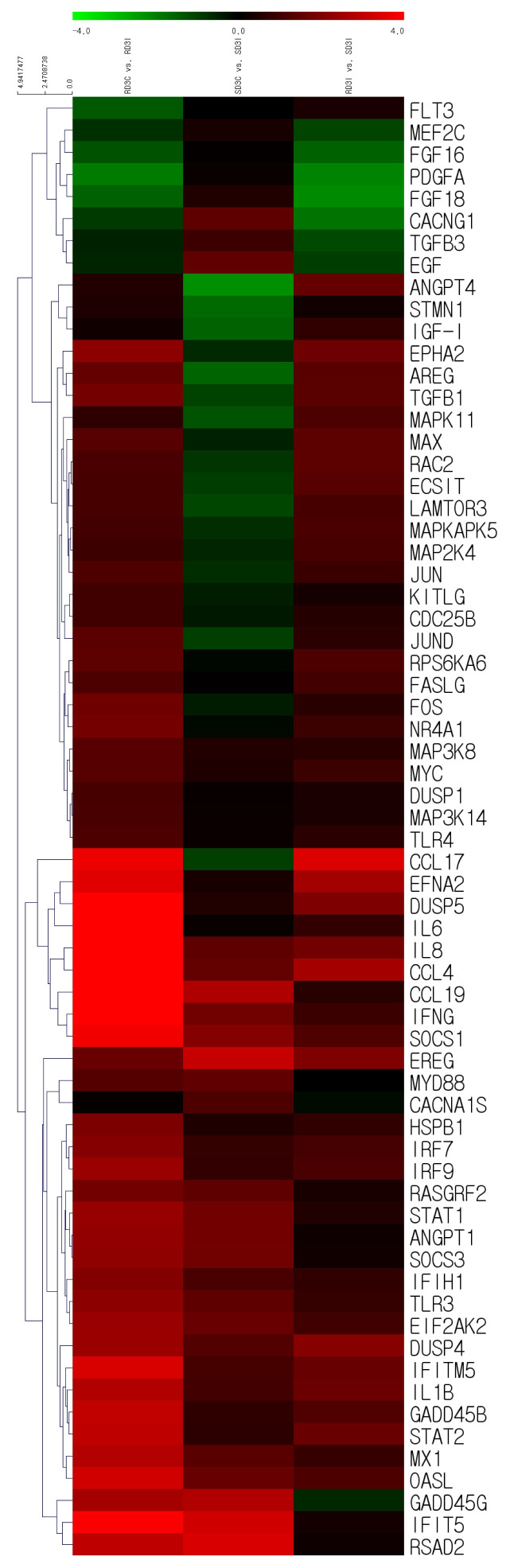
Heat map of the individual MAPK signaling pathway genes from the tracheal tissue of three comparisons of Ri chickens, three days post-infection: Control resistant vs infected resistant chickens, Control susceptible vs infected susceptible chickens, infected susceptible vs infected resistant chickens. A green color indicates DEGs that have higher expression levels while a red color indicates DEGs that have lower expression levels, as calculated from the expression values in |log_2_| (fold change) units. MAPK, mitogen-activated protein kinase; DEGs, differentially expressed genes.

**Figure 4:**
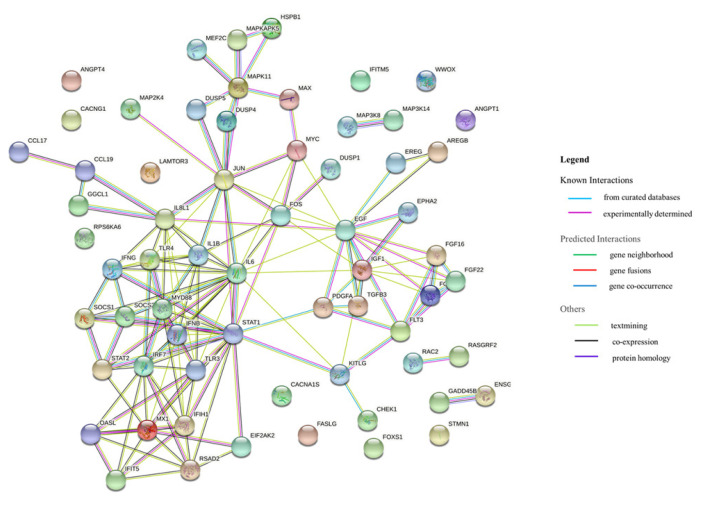
Interactions of the 68 differentially expressed genes related to the MAPK signaling pathway in the tracheas of Ri chicken lines. This interaction analysis was conducted using the STRING version 11.0 (http://string-db.org/). MAPK, mitogen-activated protein kinase.

Based on the RNA sequencing results and subsequent mapping to the KEGG pathway database, 69 DEGs were related to the MAPK signaling pathway in the three comparisons (Supplementary Table S7). Among them, 61 genes were upregulated, and 8 genes were downregulated after HPAIV-H5N1 infection in the resistant Ri chicken line. A total of 47 genes were upregulated, and 21 genes were downregulated after HPAIV-H5N1 infection in the susceptible Ri chicken line; 59 genes were upregulated and 10 genes were downregulated in the HPAI-resistant compared to the HPAI-susceptible group. Hierarchical clustering based on the expression patterns is shown in a heatmap in Figure 3. The STRING protein–protein interaction database was used for interaction analysis (Figure 4). The network contains 64 nodes with 143 edges (vs 15 expected edges), a clustering coefficient of 0.602, and an enrichment p-value<1.0e–16. The confidence score threshold was set at 0.7 (high) for analysis.

### Quantitative real-time PCR analysis of genes associated with MAPK signaling pathway

**Figure 5:**
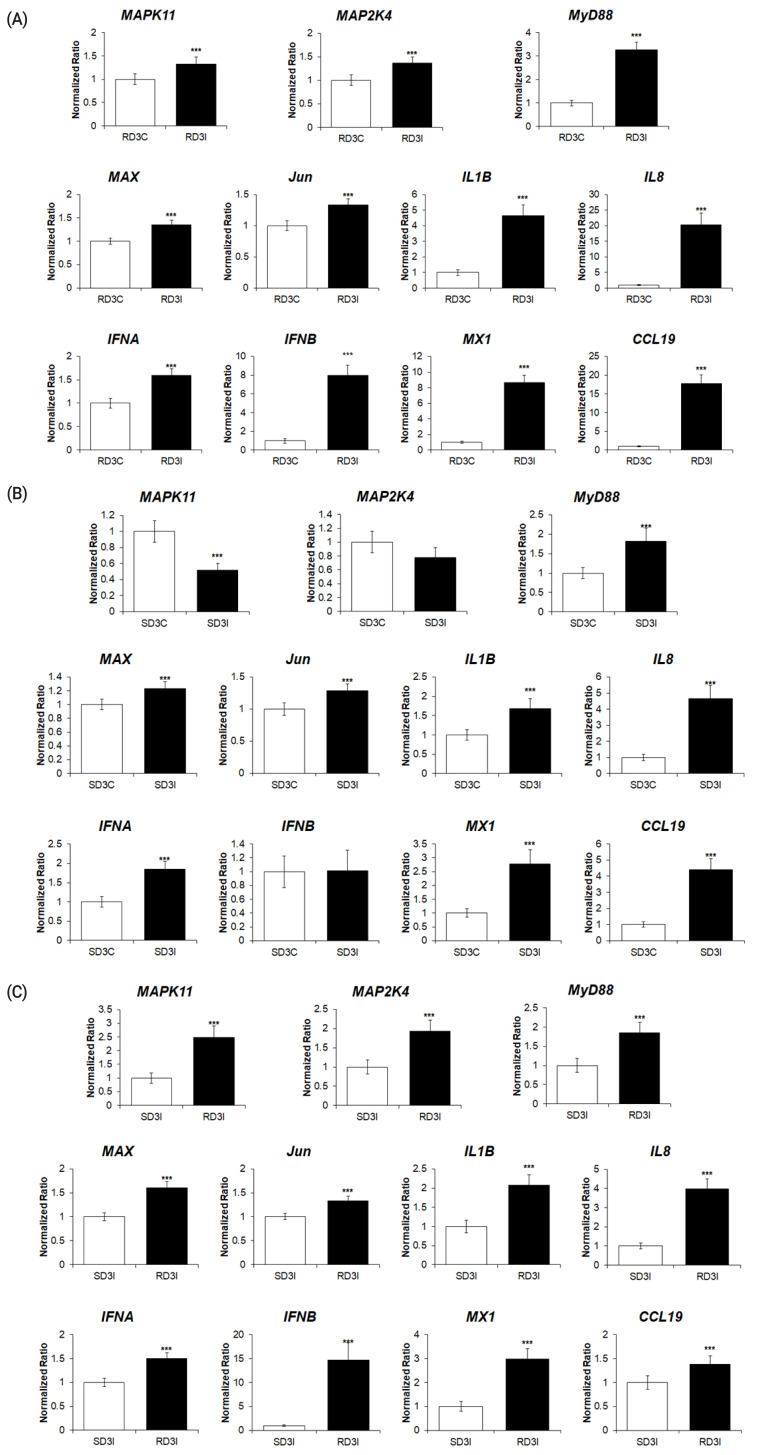
The qPCR analysis of the expression of genes associated with MAPK signaling pathway in (A) control and H5N1-infected resistant Ri chickens at 3 days post infection (dpi); (B) control and H5N1-infected susceptible Ri chickens; (C) H5N1-infected susceptible and H5N1-infected resistant Ri chickens. Relative quantitation data of qRT-PCR are represented as mean±SEM, normalized to GAPDH using the 2^−^^ΔΔ^^Ct^ method. Data are expressed as mean±SEM of three independent experiments: *** p<0.001. qPCR, real-time quantitative polymerase chain reaction; MAPK, mitogen-activated protein kinase; SEM, standard error of mean; GAPDH, glyceraldehyde-3-phosphate dehydrogenase.

**Figure 6:**
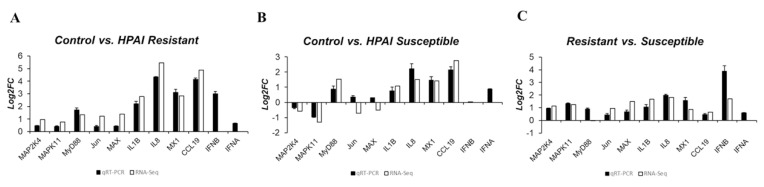
The expression patterns of these genes were compared between qPCR and RNA sequencing data (|log_2_|-fold change). The qRT-PCR data are expressed as mean±SEM of three independent experiments. qRT-PCR, real-time quantitative polymerase chain reaction; SEM, standard error of mean.

To validate the RNA sequencing results, the expression levels of 11 genes of the DEGs associated with the MAPK signaling pathway were quantitatively determined via qRT-PCR (Figure 5A–C). The expression levels of cytokines (IL-1β, IL-8), type I interferons, IFN-stimulated genes (*IFN-α*, *IFN-β*, *CCL19*, and *Mx1*), MyD88, and MAPK transcription factors (MyD88, MAP2K4, MAPK11, Jun, and MAX) were significantly upregulated after HPAIV infection in resistant chickens, and in HPAI-resistant cells compared to HPAI-susceptible cells (Figure 5A, C). The expression levels of cytokines (IL-1β, IL-8), interferon alpha, IFN-stimulated genes (*IFN-α*, *CCL19*, and *Mx1*), MyD88, MAX, and Jun were significantly upregulated after HPAIV infection in susceptible chickens. The expression levels of MAP2K4 and IFN-β were not significant, and MAPK11 was significantly downregulated (Figure 5B). These results demonstrated that the expression levels of these genes obtained by qRT-PCR were consistent with the RNA-seq results (Figure 6A–C)

## DISCUSSION

In this study, we analyzed the transcriptome profiles of control and Ri chickens infected with HPAIV H5N1 using RNA sequencing. Susceptible H5N1-infected Ri chickens and resistant H5N1-infected Ri chickens were selected based on their *Mx* and *BF2* genotypes and were infected with HPAIV H5N1. RNA sequencing was conducted after infection, and 1,794 DEGs were identified between resistant control and resistant H5N1-infected Ri chickens, 432 DEGs were identified between susceptible control and susceptible H5N1-infected Ri chickens, and 1,202 DEGs were identified after comparing the transcriptome profiles of tracheal tissue obtained from resistant and susceptible H5N1-infected chickens. KEGG analysis revealed that most of the DEGs were related to the MAPK signaling pathway.

Avian influenza viral pathogen-associated molecular pat terns are recognized by host pattern recognition receptors (PRRs). The toll-like receptor 3 (TLR3) and melanoma differentiation-associated protein 5 (MDA5, interferon induced with helicase C domain 1 [IFIH1]) response to double-stranded RNA (dsRNA) during AIV infection in chickens [19] is through adaptor protein TIR-domain-containing adapter-inducing interferon [20]. These adaptors activate the transcription factor interferon regulatory factor 7 (IRF7), or the MAPK signaling pathway activates nuclear factor kappa B (NF-κB) to induce cytokines, chemokines, and type I interferons (IFN-α and IFN-β) [21]. Our results showed that the expression levels of TLR3, IFIH1, and IRF7 were increased after infection in resistant chickens (Supplementary Table S7); and expression levels of IRF7 were higher in resistant H5N1-infected chickens than in susceptible H5N1-infected Ri chickens (Supplementary Table S7). The expression of type I interferons can be regulated by MAPK signaling pathway genes. JNK regulates IFN expression through the AP-1 transcription factor in response to influenza A virus infection [11]. The p38 kinase activation has also been shown to an essential role in cytokines, chemokines, type I interferons induction in primary human macrophages by H5N1 [22]. In addition, the gene expression in the MAPK signaling pathway in our study (e.g., *MyD88*, *AP-1*, *c-fos*, *Jun*, *JunD*, *MAX*), and cytokines (IL-1β, IL-6, IL-8) were increased after infection in resistant chickens, and the expression levels of MAP2K4 and MAPK11 were higher in resistant H5N1-infected chickens than in susceptible H5N1-infected Ri chickens (Supplementary Table S7). Type I interferons trigger the expression of IFN-stimulated genes, which block virus entry into the host cells [23]. Furthermore, p38 kinase is also able to control the expression of IFN-stimulated genes through the phosphorylation of Tyr701 and Ser727 in STAT1 [12]. High expression levels of PRRs, MyD88, IRF7, STAT1, cytokines and IFN-stimulated genes were previously observed in H5N1-infected chickens [24]. Therefore, we suggest that HPAIV H5N1 in resistant Ri chickens induces cytokines, IFNs, and IFN-stimulated genes through the MAPK signaling pathway to activate antiviral activity.

Type I interferons trigger the expression of IFN-stimulated genes through the Jak-STAT signaling cascade [23]. IFNs and IFN-stimulated genes can inhibit viral replication by blocking virus entry into the host cells, binding to viral RNA to stop translation, and regulating host antiviral responses [25]. Moreover, several studies have shown that IFN-stimulated genes have antiviral activity [26,27]. The *Mx* gene inhibits the trafficking and activity of viral polymerases [4]. Viperin (RSAD2) inhibits newly synthesized influenza virions [28]. Chicken interferon-inducible 2′-5′-oligoadenylate synthase-like (OASL) and RNase L restrict both viral and cellular RNA, preventing viral genome replication [29]. Wild-type duck OASL inhibits the replication of a variety of RNA viruses *in vitro*, including influenza virus [30]. Protein kinase R (EIF2AK2) inhibits the translation of viral mRNAs, including those from influenza A viruses [26]. Interferon-induced proteins of the tetratricopeptide repeats (IFIT) protein family sequester viral nucleic acids [31]. Furthermore, the clinical results were enhanced in chIFIT5-transgenic chickens after treatment with HPAIV and Newcastle disease virus [27]. Our results showed a higher expression of IFNs, STAT1, and IFN-stimulated genes (*Mx*, *CCL19*, *OASL*, *RSAD2*, *EIF2AK2*, *IFITM5*, and *IFIT5*) were increased after infection in resistant chickens, and the expression levels of STAT2, SOCS1, OASL, and EIF2AK2 were higher in H5N1-infected than in susceptible H5N1-infected Ri chickens (Supplementary Table S7). Furthermore, IFN-α and IFN-β expression was increased in H5N1-infected resistant Ri chickens in qRT-PCR results and in H5N1-infected resistant chickens compared to H5N1-infected susceptible Ri chickens. Therefore, we suggest that resistant Ri chickens have an antiviral response to HPAIV H5N1, and resistant Ri chickens have an antiviral response higher than susceptible chickens.

In summary, the 4-week-old Ri chickens were infected with H5N1 HPAIV and the chickens had ruffled hair and tracheal hemorrhage. To clarify the gene expression after H5N1 infection between two chicken lines, we evaluated the differential expression of genes related to the MAPK signaling pathway in the tracheal tissues of three comparison groups: susceptible control vs infection, resistant control vs infection, and resistant infection vs. susceptible infection after three days of H5N1 infection, using RNA sequencing and quantitative real-time PCR. Interestingly, the expression of PRRs, MAPK signaling pathway genes (*MyD88*, *AP-1*, *c-fos*, *Jun*, *JunD*, MAX, and *c-Myc*), cytokines, chemokines, IFNs, and IFN-stimulated genes were increased after infection in resistant chickens. MyD88, Jun, JunD, MAX, cytokines, chemokines, IFNs, and IFN-stimulated expressed genes were higher in resistant H5N1-infected than in susceptible H5N1-infected Ri chickens. These results suggest that resistant Ri chickens show higher antiviral activity compared to susceptible Ri chickens, and antiviral activity through the MAPK signaling pathway activates antiviral genes in H5N1-infected resistant Ri chickens. This resistant Ri chicken (*Mx*/A; BF2/B21) is considered a potential HPAIV-resistant chicken line, and further studies are necessary to understand the immune mechanisms of defense against HPAIV.

## Figures and Tables

**Table 1:** Primer sequences used for quantitative real-time polymerase chain reaction

Genes		Primer sequence	Product (bp)	GenBank accession No.
*GAPDH*	F	TGC TGC CCA GAA CAT CAT CC	142	NM_204305
	R	ACG GCA GGT CAG GTC AAC AA		
*IL-1* *β*	F	TGC CTG CAG AAG AAG CCT CG	137	NM_204524.1
	R	CTC CGC AGC AGT TTG GTC AT		
*IL-6*	F	GCA GGA CGA GAT GTG CAA GA	131	NM_204628.1
	R	ATT TCT CCT CGT CGA AGC CG		
*IL-8*	F	GGC TTG CTA GGG GAA ATG A	200	NM_205498.1
	R	AGC TGA CTC TGA CTA GGA AAC TGT		
*Jun*	F	CGC GGG CTC TGT TCT ATG	118	NM_001031289
	R	TCA GCA CCT TGG CGT TAT TAT		
*MAX*	F	TGG AGA GCG ACG AGG AG	174	XM_015286092.2
	R	CTT TGT CCA GGA TTT GGG C		
*MyD88*	F	CGG CTG ATT CCG GTC AAG TG	142	NM_001030962.4
	R	ATC ACG GCA GCA AGA GAG AT		
*IFN-* *α*	F	GAG CAA TGC TTG GAC AGC AG	183	GU119896.1
	R	GAG GTT GTG GAT GTG CAG GA		
*IFN-* *β*	F	CTT GCC CAC AAC AAG ACG TG	139	NM_001024836.1
	R	TGT TTT GGA GTG TGT GGG CT		
*MX1*	F	AGC CAT AGA ACA AGC CAG AA	127	NM_204609.1
	R	GGT ACT GGT AAG GAA GGT GG		
*MAPK11*	F	TCC GCT AAA ATG TCC GAG C	134	NM_001006227.1
	R	TCA TAA GCT GAA CAC ACG GA		
*MAP2K4*	F	CCA AAA ATA TGT CGC GTT GA	117	XM_015295240.2
	R	TAC AGG ACG CCT AGT TAA GA		

*GAPDH*, glyceraldehyde-3-phosphate dehydrogenase; *IL-1β*, interleukin-1β; *IL-6*, interleukin-6; *IL-8*, interleukin-8; *Jun*, AP-1 transcription factor subunit; *MAX*, MYC associated factor X; *MyD88*, myeloid differentiation primary response 88; *IFN-α*, interferons; *IFN-β*, interferons; *MX1*, MX dynamin like GTPase 1; *MAPK11*, mitogen-activated protein kinase 11; *MAP2K4*, mitogen-activated protein kinase kinase 4.

## SUPPLEMENTARY MATERIAL

**Supplementary Figure S1**. Gene ontology (GO) functional analysis for control and HPAI-infected resistant Ri chicken lines. Supplementary Figure S2. Gene ontology (GO) functional analysis for control and HPAI infected susceptible Ri chicken lines. **Supplementary Table S1**. Number of Ri chicken samples in each group **Supplementary Table S2**. Summary of raw reads and clean reads of trachea tissue samples obtained from the control and highly pathogenic avian influenza virus-infected chickens from 3 days post-infection (dpi) datasets. **Supplementary Table S3**. Sequence alignment of the control and highly pathogenic avian influenza virus-infected samples at 3 days post-infection **Supplementary Table S4**. Gene ontology enrichment of sequence data obtained from the tracheal tissue of control and H5N1-infected resistant chickens, with p<0.05 and |Log_2_|-transformation of the normalization of H5N1-infected/control resistant chickens ≥2. **Supplementary Table S5**. Gene ontology enrichment of sequence data obtained from the tracheal tissue of control and H5N1-infected susceptible chickens, with p<0.05 and |Log_2_|-transformation of the normalization of H5N1-infected/control susceptible chickens ≥2. **Supplementary Table S6**. Gene ontology enrichment of sequence data obtained from the tracheal tissue of susceptible and H5N1-infected resistant chickens, with p<0.05 and |Log_2_|-transformation of the normalization of resistant/susceptible ≥2. **Supplementary Table S7**. The 69 differentially expressed genes (DEGs) related to the MAPK signaling pathway among the control, H5N1-infected resistant, and susceptible Ri chickens in the trachea (p-value).
