# Supplementary material for: The highly pathogenic H5N1 avian influenza virus induces the mitogen-activated protein kinase signaling pathway in the trachea of two Ri chicken lines
Source: Anim Biosci. 2022 Jan 5;35(7):964–74. doi: 10.5713/ab.21.0420 (PMC9271386; doi:10.5713/ab.21.0420)
Supplement: Supplementary file 1 [file ab-21-0420-suppl.pdf]

Figure S1. Gene ontology (GO) functional analysis for control and HPAI-infected resistant Ri chicken lines. The enriched biological terms include: **(A)** Top 10 singular enrichment analysis terms (SEA): GO biological process, **(B)** GO cellular component, and **(C)** GO molecular function **(D)** KEGG functional pathways from the 1794 DEGs in control and HPAI-infected resistant Ri chicken lines obtained by criteria (|Fold-Change| ≥ 2) ∩ ($p < 0.05$)

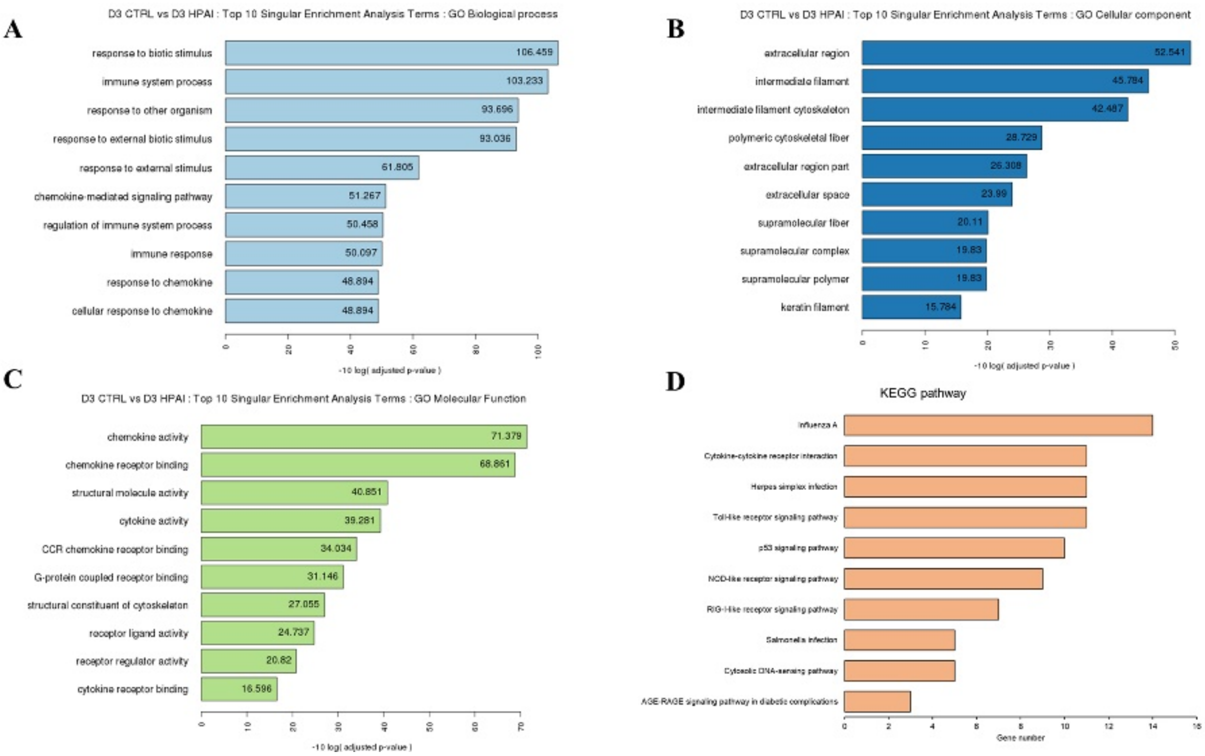

Figure S2. Gene ontology (GO) functional analysis for control and HPAI infected susceptible Ri chicken lines. The enriched biological terms include: **(A)** Top 10 singular enrichment analysis terms (SEA): GO biological process, **(B)** GO cellular component, and **(C)** GO molecular function **(D)** KEGG functional pathways from the 432 DEGs in control and HPAI infected susceptible Ri chicken lines obtained by criteria (|Fold-Change| ≥ 2) ∩ ($p < 0.05$)

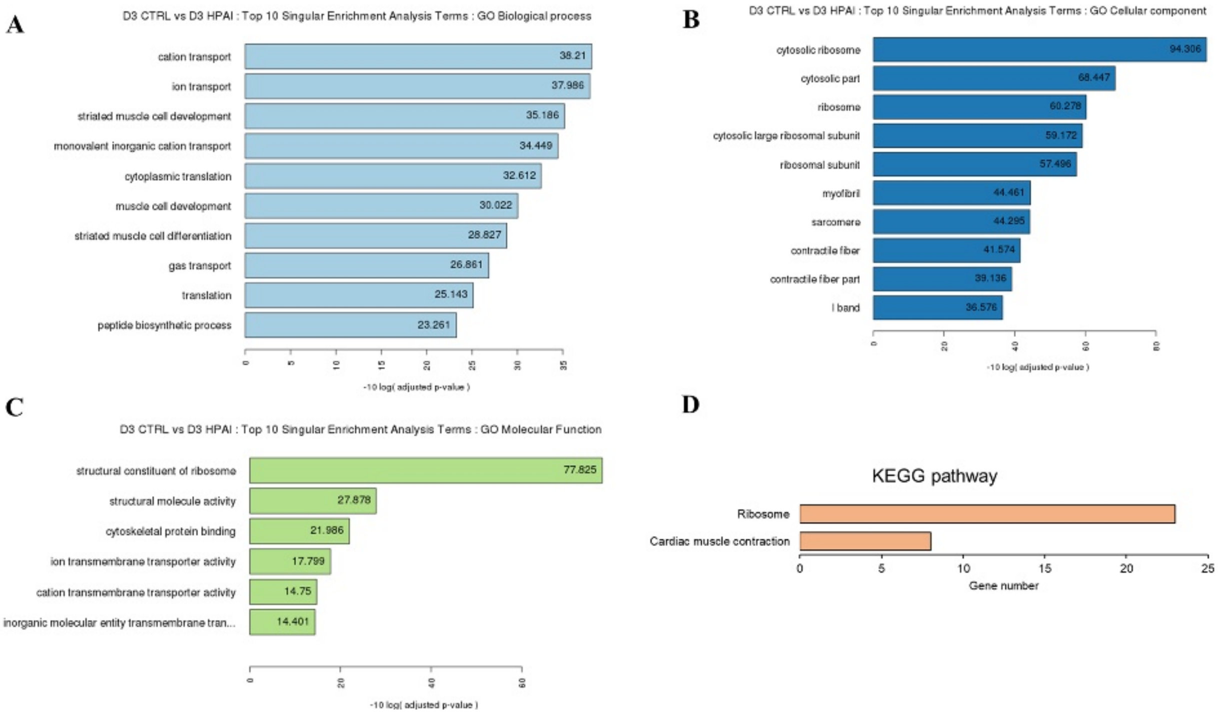

Table S1. Number of Ri chicken samples in each group

Sample	Genotype											
Ri chicken (60)	Resistant (*Mx*/A; *BF2*/B21)						Susceptible (*Mx*/G; *BF2*/B13)					
	Control			HPAI treatment			Control			HPAI treatment		
	Day 1	Day 2	Day 3	Day 1	Day 2	Day 3	Day 1	Day 2	Day 3	Day 1	Day 2	Day 3
	5	5	5	5	5	5	5	5	5	5	5	5

Table S2. Summary of raw reads and clean reads of trachea tissue samples obtained from the control and highly pathogenic avian influenza virus-infected chickens from 3 dpi datasets

Sample name	Sample group	Raw Reads				Clean Reads			
		# Read pairs	Yield (bp)	*% ≥ Q30 Bases	Mean	# Read pairs	Yield (bp)	*% ≥ Q30 Bases	Mean
R1D3C	Resistant control	21,548,249	6,464,474,700	89.0	Q34.7	21,548,147	6,442,530,524	89.1	Q34.8
R2D3C	Resistant control	17,908,380	5,372,514,000	88.7	Q34.7	17,907,998	5,336,872,496	88.9	Q34.7
R4D3C	Resistant control	19,259,603	5,777,880,900	90.3	Q35.0	19,259,306	5,720,820,592	90.6	Q35.1
R1D3I	Resistant infection	31,611,056	9,483,316,800	90.1	Q35.0	31,610,873	9,425,678,618	90.3	Q35.0
R2D3I	Resistant infection	28,566,529	8,569,958,700	89.6	Q34.8	28,566,381	8,520,869,640	89.7	Q34.9
R3D3I	Resistant infection	26,453,085	7,935,925,500	90.6	Q35.1	26,452,941	7,885,140,920	90.8	Q35.1
R5D3I	Resistant infection	28,023,145	8,406,943,500	91.0	Q35.2	28,022,888	8,352,819,070	91.1	Q35.2
S2D3C	Susceptible control	18,343,874	5,503,162,200	88.3	Q34.5	18,343,740	5,459,766,476	88.6	Q34.6
S3D3C	Susceptible control	19,947,065	5,984,119,500	89.8	Q34.9	19,946,922	5,955,753,754	90.0	Q34.9
S1D3I	Susceptible infection	22,861,780	6,858,534,000	89.0	Q34.7	22,861,601	6,801,971,322	89.3	Q34.8
S2D3I	Susceptible infection	18,937,511	5,681,253,300	89.1	Q34.7	18,937,324	5,643,362,480	89.3	Q34.8
S3D3I	Susceptible infection	19,357,823	5,807,346,900	89.5	Q34.8	19,357,670	5,775,981,908	89.7	Q34.8
S5D3I	Susceptible infection	19,099,583	5,729,874,900	89.4	Q34.8	19,099,480	5,701,878,192	89.6	Q34.8

Table S3. Sequence alignment of the control and highly pathogenic avian influenza virus-infected samples at 3 dpi

Sample name	Sample group	Mapping rate (%)	Unique mapped	Multiple mapped	Total unmapped	Unmapped by too short alignment (%)
R1D3C	Resistant control	92.66	19,469,728	497,060	1,581,359	6.72
R2D3C	Resistant control	87.71	15,203,848	504,109	2,200,041	11.63
R4D3C	Resistant control	91.61	17,224,754	418,561	1,615,991	8.01
R1D3I	Resistant infection	90.27	21,954,051	727,226	2,445,662	9.05
R2D3I	Resistant infection	84.35	23,097,588	997,941	4,470,852	14.93
R3D3I	Resistant infection	91.73	23,574,964	691,357	2,186,620	7.57
R5D3I	Resistant infection	91.34	24,993,539	602,831	2,426,518	8.08
S2D3C	Susceptible control	83.83	14,268,198	1,109,800	2,965,742	15.57
S3D3C	Susceptible control	90.60	17,696,993	374,882	1,875,047	9.02
S1D3I	Susceptible infection	91.53	20,368,414	557,492	1,935,695	7.75
S2D3I	Susceptible infection	89.29	16,488,013	421,855	2,027,456	10.23
S3D3I	Susceptible infection	92.62	17,527,918	400,867	1,428,885	7.02
S5D3I	Susceptible infection	92.43	17,255,632	398,507	1,445,341	6.85

Table S4. Gene ontology and Kyoto Encyclopedia of Genes and Genomes enrichment of control versus HPAI-infected resistant Ri chicken in trachea for DEGs with $p < 0.05$ and Log2-transformation of the normalization of control/infected ≥2 (1)

Domain	Term ID	Term name	Gene number	p-value
BP	GO:0008544	epidermis development	17	0.00437
BP	GO:0009913	epidermal cell differentiation	10	0.0158
BP	GO:0043588	skin development	15	0.0182
BP	GO:0044070	regulation of anion transport	6	0.0441
BP	GO:0042130	negative regulation of T cell proliferation	7	0.0271
BP	GO:0046007	negative regulation of activated T cell proliferation	4	0.00724
BP	GO:0015698	inorganic anion transport	10	0.0194
BP	GO:0018149	peptide cross-linking	4	0.000809
BP	GO:0001816	cytokine production	33	0.0175
BP	GO:0001817	regulation of cytokine production	31	0.00853
BP	GO:0044341	sodium-dependent phosphate transport	3	0.0368
BP	GO:0030851	granulocyte differentiation	6	0.0129
BP	GO:0008283	cell proliferation	89	0.0133
BP	GO:0042127	regulation of cell proliferation	75	0.0238
BP	GO:0002376	immune system process	94	4.75E-11
BP	GO:0002253	activation of immune response	21	0.0244
BP	GO:0002218	activation of innate immune response	13	0.0489
BP	GO:0002682	regulation of immune system process	55	0.000009
BP	GO:0002683	negative regulation of immune system process	20	0.00857
BP	GO:0002684	positive regulation of immune system process	42	3.95E-05
BP	GO:0051704	multi-organism process	50	0.0207
BP	GO:0050896	response to stimulus	281	0.0208
BP	GO:0042221	response to chemical	103	0.000301
BP	GO:0010033	response to organic substance	64	0.000743
BP	GO:0034097	response to cytokine	30	1.57E-05
BP	GO:1990868	response to chemokine	7	1.29E-05
BP	GO:0070555	response to interleukin-1	6	0.000448
BP	GO:0009605	response to external stimulus	68	6.6E-07
BP	GO:0009607	response to biotic stimulus	39	2.26E-11
BP	GO:0043207	response to external biotic stimulus	36	4.97E-10

BP	GO:0051707	response to other organism	36	4.27E-10
BP	GO:0009617	response to bacterium	20	0.000448
BP	GO:0009615	response to virus	16	0.000272
BP	GO:0002237	response to molecule of bacterial origin	17	0.00637
BP	GO:0032496	response to lipopolysaccharide	13	0.015
BP	GO:0006955	immune response	46	9.78E-06
BP	GO:0050776	regulation of immune response	34	0.00662
BP	GO:0070887	cellular response to chemical stimulus	70	2.11E-05
BP	GO:0071310	cellular response to organic substance	56	0.00144
BP	GO:0071345	cellular response to cytokine stimulus	25	0.00216
BP	GO:0071347	cellular response to interleukin-1	5	0.00671
BP	GO:1990869	cellular response to chemokine	7	1.29E-05
BP	GO:0001775	cell activation	32	0.00746
BP	GO:0045321	leukocyte activation	30	0.00314
BP	GO:0006952	defense response	55	2.29E-05
BP	GO:0045087	innate immune response	31	6.09E-05
BP	GO:0034341	response to interferon-gamma	5	0.0405
BP	GO:0071346	cellular response to interferon-gamma	5	0.0275
BP	GO:0098542	defense response to other organism	17	0.00273
BP	GO:0002520	immune system development	30	0.00671
BP	GO:0048534	hematopoietic or lymphoid organ development	29	0.00564
BP	GO:0030097	hemopoiesis	28	0.00442
BP	GO:0050900	leukocyte migration	10	0.00012
BP	GO:0097529	myeloid leukocyte migration	6	0.0162
BP	GO:0097530	granulocyte migration	6	0.00118
BP	GO:1990266	neutrophil migration	6	0.000353
BP	GO:1902622	regulation of neutrophil migration	3	0.0174
BP	GO:1902624	positive regulation of neutrophil migration	3	0.00701
BP	GO:0072676	lymphocyte migration	5	0.00423
BP	GO:0071674	mononuclear cell migration	5	0.0033
BP	GO:0002523	leukocyte migration involved in inflammatory response	2	0.0178
BP	GO:0060326	cell chemotaxis	21	0.00382
BP	GO:0030595	leukocyte chemotaxis	8	0.000739
BP	GO:0048247	lymphocyte chemotaxis	5	0.000226
BP	GO:0071621	granulocyte chemotaxis	6	0.000564
BP	GO:0030593	neutrophil chemotaxis	6	0.00016

BP	GO:0002548	monocyte chemotaxis	5	0.00104
BP	GO:0002232	leukocyte chemotaxis involved in inflammatory response	3	0.00188
BP	GO:0019221	cytokine-mediated signaling pathway	20	8.08E-05
BP	GO:0070098	chemokine-mediated signaling pathway	7	7.47E-06
BP	GO:0071622	regulation of granulocyte chemotaxis	3	0.0308
BP	GO:0090022	regulation of neutrophil chemotaxis	3	0.0125
BP	GO:0071624	positive regulation of granulocyte chemotaxis	3	0.00701
BP	GO:0090023	positive regulation of neutrophil chemotaxis	3	0.00561
CC	GO:0005576	extracellular region	85	5.57E-06
CC	GO:0044421	extracellular region part	64	0.00234
CC	GO:0005615	extracellular space	14	0.00399
CC	GO:0099080	supramolecular complex	10	0.0104
CC	GO:0099081	supramolecular polymer	10	0.0104
CC	GO:0099512	supramolecular fiber	10	0.00975
CC	GO:0099513	polymeric cytoskeletal fiber	10	0.00134
CC	GO:0045111	intermediate filament cytoskeleton	8	5.64E-05
CC	GO:0005882	intermediate filament	8	2.64E-05
CC	GO:0045095	keratin filament	6	0.0264
MF	GO:0030545	receptor regulator activity	28	0.00828
MF	GO:0001664	G-protein coupled receptor binding	11	0.000768
MF	GO:0005126	cytokine receptor binding	7	0.0219
MF	GO:0042379	chemokine receptor binding	7	1.3E-07
MF	GO:0048020	CCR chemokine receptor binding	4	0.000395
MF	GO:0048018	receptor ligand activity	28	0.00336
MF	GO:0005125	cytokine activity	8	0.000118
MF	GO:0008009	chemokine activity	7	7.28E-08
MF	GO:0005198	structural molecule activity	12	8.22E-05
MF	GO:0005200	structural constituent of cytoskeleton	6	0.00197
MF	GO:0005153	interleukin-8 receptor binding	2	0.0255
keg	KEGG:05168	Herpes simplex infection	11	0.00364
keg	KEGG:04620	Toll-like receptor signaling pathway	11	0.00391
keg	KEGG:04115	p53 signaling pathway	10	0.0298
keg	KEGG:04621	NOD-like receptor signaling pathway	9	0.0222
keg	KEGG:04933	AGE-RAGE signaling pathway in diabetic complications	3	0.0168
keg	KEGG:05164	Influenza A	14	0.0000058
keg	KEGG:04622	RIG-I-like receptor signaling pathway	7	0.00363
keg	KEGG:05132	Salmonella infection	5	0.00707

keg	KEGG:04623	Cytosolic DNA-sensing pathway	5	0.0475
keg	KEGG:04060	Cytokine-cytokine receptor interaction	11	0.0000634

(1) Log2(FC): For a gene, calculated by ‘Log2(Experiment) - Log2(Control)’. ‘(Experiment)’ means a mean FPKM of experimental group, and ‘(Control)’ means a mean FPKM of control group. MF: molecular function, BP: biological process, and CC: cellular component.

Table S5. Gene ontology and Kyoto Encyclopedia of Genes and Genomes enrichment of control versus infected susceptible Ri chicken in trachea for DEGs with $p < 0.05$ and Log2-transformation of the normalization of control/infected ≥2 [(1)]

Domain	Term ID	Term name	Gene number	p-value
BP	GO:0006518	peptide metabolic process	26	0.0448
BP	GO:0043604	amide biosynthetic process	27	0.00809
BP	GO:0043043	peptide biosynthetic process	25	0.00472
BP	GO:0006412	translation	25	0.00306
BP	GO:0002181	cytoplasmic translation	10	0.000548
BP	GO:0042692	muscle cell differentiation	12	0.027
BP	GO:0051146	striated muscle cell differentiation	12	0.00131
BP	GO:0055001	muscle cell development	10	0.000995
BP	GO:0055002	striated muscle cell development	10	0.000303
BP	GO:0030239	myofibril assembly	7	0.0329
BP	GO:0051179	localization	106	0.0388
BP	GO:0051234	establishment of localization	86	0.0317
BP	GO:0006810	transport	84	0.0337
BP	GO:0015669	gas transport	4	0.00206
BP	GO:0006811	ion transport	46	0.000159
BP	GO:0006812	cation transport	36	0.000151
BP	GO:0015672	monovalent inorganic cation transport	24	0.000359
BP	GO:0032879	regulation of localization	53	0.0173
BP	GO:0051049	regulation of transport	38	0.0271
BP	GO:0043269	regulation of ion transport	19	0.0326
BP	GO:0034220	ion transmembrane transport	35	0.0239
BP	GO:0098655	cation transmembrane transport	28	0.00476
CC	GO:0032991	protein-containing complex	106	0.0151
CC	GO:0043228	non-membrane-bounded organelle	91	0.000641
CC	GO:0043232	intracellular non-membrane-bounded organelle	91	0.000641
CC	GO:0044445	cytosolic part	20	0.000000143
CC	GO:0005840	ribosome	20	0.000000938
CC	GO:0042788	polysomal ribosome	6	0.00105
CC	GO:0022626	cytosolic ribosome	17	3.71E-10

CC	GO:0044391	ribosomal subunit	18	0.00000178
CC	GO:0015934	large ribosomal subunit	12	0.000734
CC	GO:0022625	cytosolic large ribosomal subunit	11	0.00000121
CC	GO:0043292	contractile fiber	15	0.0000696
CC	GO:0044449	contractile fiber part	14	0.000122
CC	GO:0030016	myofibril	15	0.0000358
CC	GO:0030017	sarcomere	14	0.0000372
CC	GO:0031674	I band	11	0.00022
MF	GO:0015075	ion transmembrane transporter activity	33	0.0166
MF	GO:0008324	cation transmembrane transporter activity	24	0.0335
MF	GO:0005198	structural molecule activity	34	0.00163
MF	GO:0003735	structural constituent of ribosome	20	1.65E-08
MF	GO:0015318	inorganic molecular entity transmembrane transporter activity	31	0.0363
MF	GO:0008092	cytoskeletal protein binding	31	0.00633
keg	KEGG:04530	Tight junction	10	0.012
keg	KEGG:03010	Ribosome	20	0.0000447
keg	KEGG:04260	Cardiac muscle contraction	8	0.012

(1) Log2(FC): For a gene, calculated by 'Log2(Experiment) - Log2(Control)'. '(Experiment)' means a mean FPKM of experimental group, and '(Control)' means a mean FPKM of control group. MF: molecular function, BP: biological process, and CC: cellular component.

Table S6. Gene ontology and Kyoto Encyclopedia of Genes and Genomes enrichment of infected resistant versus infected susceptible Ri chicken in trachea for DEGs with $p < 0.05$ and Log2-transformation of the normalization of resistant/susceptible ≥2 [(1)]

Domain	Term ID	Term name	Gene number	p-value
BP	GO:0002232	leukocyte chemotaxis involved in inflammatory response	3	0.0141
BP	GO:0009605	response to external stimulus	92	0.00175
BP	GO:0006952	defense response	57	0.032
BP	GO:0045104	intermediate filament cytoskeleton organization	7	0.0472
BP	GO:0010951	negative regulation of endopeptidase activity	6	0.0488
BP	GO:0009888	tissue development	84	0.00194
BP	GO:0008544	epidermis development	11	0.0217
BP	GO:0048513	animal organ development	125	0.00538
BP	GO:0043588	skin development	10	0.044
BP	GO:0002376	immune system process	101	0.000156
BP	GO:0014706	striated muscle tissue development	27	0.0438
BP	GO:0032502	developmental process	196	0.0307
BP	GO:0009617	response to bacterium	30	0.00852

CC	GO:0005576	extracellular region	40	0.000432
CC	GO:0044421	extracellular region part	25	0.00913
CC	GO:0005615	extracellular space	26	0.00881
CC	GO:0009986	cell surface	30	0.0419
CC	GO:0030057	desmosome	6	0.0144
CC	GO:0045111	intermediate filament cytoskeleton	7	0.00466
CC	GO:0005882	intermediate filament	7	0.00243
CC	GO:0045095	keratin filament	5	0.0288
MF	GO:0005198	structural molecule activity	12	0.0138
keg	KEGG:00140	Steroid biosynthesis	5	0.07607
keg	KEGG:00030	Pentose phosphate pathway	7	0.00814
keg	KEGG:04916	Melanogenesis	12	0.08014
keg	KEGG:04310	Wnt signaling pathway	14	0.09420
keg	KEGG:03010	Ribosome	20	0.02178
keg	KEGG:04010	MAPK signaling pathway	24	0.04357

(1) Log2(FC): For a gene, calculated by 'Log2(Experiment) - Log2(Control)'. '(Experiment)' means a mean FPKM of experimental group, and '(Control)' means a mean FPKM of control group. MF: molecular function, BP: biological process, and CC: cellular component.

Table S7: 69 DEGs related to the MAPK signaling pathway between control, H5N1-infected resistant and susceptible Ri chickens in trachea (*p*-value).

**Gene name**	**RD3C vs. RD3I**		**SD3C vs. SD3I**		**RD3I vs. SD3I**		**Full descriptions of the gene**
	**Log2(FC)**	***p*-value**	**Log2(FC)**	***p*-value**	**Log2(FC)**	***p*-value**	
EFNA2	3.550	0.026	0.416	1.000	2.586	0.028	ephrin A2 [Source:NCBI gene;Acc:395831]
DUSP4	2.453	0.011	1.314	1.000	2.141	0.008	dual specificity phosphatase 4 [Source:NCBI gene;Acc:395642]
DUSP5	4.515	0.000	0.542	0.358	2.022	0.000	dual specificity phosphatase 5 [Source:HGNC Symbol;Acc:HGNC:3071]
EREG	1.677	0.058	3.123	1.000	2.013	0.039	epiregulin [Source:NCBI gene;Acc:408036]
EPHA2	2.213	0.000	-0.654	0.231	1.737	0.000	EPH receptor A2 [Source:HGNC Symbol;Acc:HGNC:3386]
ANGPT4	0.568	0.284	-2.244	0.098	1.611	0.005	angiopoietin 4 [Source:HGNC Symbol;Acc:HGNC:487]
MAX	1.380	0.001	-0.518	0.412	1.477	0.001	MYC associated factor X [Source:HGNC Symbol;Acc:HGNC:6913]
RAC2	1.207	0.001	-0.834	0.113	1.467	0.000	ras-related C3 botulinum toxin substrate 2 (rho family, small GTP binding protein Rac2)

							[Source:NCBI gene;Acc:418044]
ECSIT	1.145	0.008	-0.963	0.121	1.375	0.007	ECSIT signalling integrator [Source:HGNC Symbol;Acc:HGNC:29548]
GADD45B	3.054	0.000	0.766	0.298	1.287	0.014	growth arrest and DNA damage inducible beta [Source:HGNC Symbol;Acc:HGNC:4096]
MAPK11	0.759	0.101	-1.302	0.065	1.239	0.026	mitogen-activated protein kinase 11 [Source:NCBI gene;Acc:417739]
RPS6KA6	1.508	0.000	-0.095	0.850	1.236	0.001	ribosomal protein S6 kinase A6 [Source:HGNC Symbol;Acc:HGNC:10435]
MAPKAPK 5	1.079	0.002	-0.731	0.194	1.177	0.003	mitogen-activated protein kinase-activated protein kinase 5 [Source:NCBI gene;Acc:373929]
MAP2K4	0.964	0.012	-0.588	0.335	1.137	0.011	mitogen-activated protein kinase kinase 4 [Source:HGNC Symbol;Acc:HGNC:6844]
LAMTOR3	1.121	0.002	-1.107	0.039	1.133	0.006	late endosomal/lysosomal adaptor, MAPK and MTOR activator 3 [Source:NCBI gene;Acc:425210]
FASLG	1.238	0.009	0.058	0.937	1.081	0.031	Fas ligand (TNF superfamily, member 6) [Source:NCBI gene;Acc:429064]
MEF2C	-0.758	0.041	0.376	0.504	-1.080	0.014	myocyte enhancer factor 2C [Source:HGNC Symbol;Acc:HGNC:6996]
TGFB3	-0.520	0.088	0.987	0.056	-1.171	0.002	transforming growth factor beta 3 [Source:NCBI gene;Acc:396438]
FGF16	-1.301	0.046	0.086	0.907	-1.525	0.027	fibroblast growth factor 16 [Source:NCBI gene;Acc:422330]
CACNG1	-0.902	0.013	1.509	0.007	-1.808	0.000	calcium voltage-gated channel auxiliary subunit gamma 1 [Source:NCBI gene;Acc:417428]
PDGFA	-1.937	0.000	0.168	0.754	-2.056	0.000	platelet derived growth factor subunit A [Source:NCBI gene;Acc:374196]
FGF18	-1.522	0.000	0.538	0.335	-2.170	0.000	fibroblast growth factor 18 [Source:NCBI gene;Acc:395453]
FGF22	4.321	0.190	-Inf	1.000	Inf	0.000	fibroblast growth factor 22 [Source:HGNC Symbol;Acc:HGNC:3679]
FLT3	-1.397	0.036	-0.014	1.000	0.432	1.000	fms related tyrosine kinase 3 [Source:HGNC Symbol;Acc:HGNC:3765]

KITLG	1.024	0.031	-0.460	0.468	0.349	0.468	KIT ligand [Source:NCBI gene;Acc:396028]
CDC25B	1.030	0.001	-0.367	0.492	0.608	0.096	cell division cycle 25B [Source:HGNC Symbol;Acc:HGNC:1726]
DUSP1	1.132	0.004	0.145	0.809	0.427	0.359	dual specificity phosphatase 1 [Source:NCBI gene;Acc:374192]
MAP3K14	1.171	0.038	0.179	0.827	0.443	0.542	mitogen-activated protein kinase kinase kinase 14 [Source:NCBI gene;Acc:419964]
JUN	1.241	0.000	-0.715	0.295	0.938	0.026	Jun proto-oncogene, AP-1 transcription factor subunit [Source:NCBI gene;Acc:424673]
MYD88	1.335	0.000	1.523	0.040	-0.007	0.989	myeloid differentiation primary response 88 [Source:NCBI gene;Acc:420420]
MAP3K8	1.377	0.000	0.571	0.340	0.651	0.109	mitogen-activated protein kinase kinase kinase 8 [Source:HGNC Symbol;Acc:HGNC:6860]
JUND	1.441	0.000	-0.989	0.152	0.693	0.103	JunD proto-oncogene, AP-1 transcription factor subunit [Source:HGNC Symbol;Acc:HGNC:6206]
MYC	1.380	0.000	0.516	0.324	0.954	0.033	v-myc avian myelocytomatosis viral oncogene homolog [Source:NCBI gene;Acc:420332]
AREG	1.604	0.024	-1.586	0.090	1.401	0.055	amphiregulin [Source:NCBI gene;Acc:428752]
FOS	1.742	0.000	-0.430	0.528	0.649	0.160	Fos proto-oncogene, AP-1 transcription factor subunit [Source:NCBI gene;Acc:396512]
RASGRF2	1.809	0.000	1.506	0.029	0.419	0.319	Ras protein specific guanine nucleotide releasing factor 2 [Source:HGNC Symbol;Acc:HGNC:9876]
TGFB1	1.857	0.043	-1.039	0.721	1.427	0.344	transforming growth factor beta 1 [Source:NCBI gene;Acc:100873157]
NR4A1	1.866	0.001	-0.144	0.831	0.948	0.043	nuclear receptor subfamily 4 group A member 1 [Source:HGNC Symbol;Acc:HGNC:7980]
HSPB1	1.987	0.000	0.511	0.383	0.779	0.076	heat shock protein family B (small) member 1 [Source:NCBI gene;Acc:396227]
ANGPT1	2.317	0.000	1.829	0.006	0.250	0.612	angiopoietin 1 [Source:NCBI gene;Acc:395129]

GADD45G	2.603	0.000	2.797	0.007	-0.620	0.340	growth arrest and DNA damage inducible gamma [Source:NCBI gene;Acc:429929]
CACNA1S	0.127	0.751	1.231	0.033	-0.172	0.699	calcium voltage-gated channel subunit alpha1 S [Source:NCBI gene;Acc:395985]
EGF	-0.554	0.177	1.531	0.015	-0.976	0.037	epidermal growth factor [Source:NCBI gene;Acc:408035]
STMN1	0.511	0.151	-1.674	0.005	0.281	0.501	stathmin 1 [Source:NCBI gene;Acc:396057]
IGF-I	0.283	0.522	-1.543	0.020	0.769	0.162	insulin like growth factor 1 [Source:NCBI gene;Acc:418090]
IL1B	2.784	0.019	1.075	1.000	1.683	0.030	interleukin 1, beta [Source:NCBI gene;Acc:395196]
IL8	5.451	0.005	1.521	0.095	1.827	0.009	interleukin 8-like 2 [Source:NCBI gene;Acc:396495]
IL8L1	Inf	0.000	0.605	1.000	1.249	0.097	interleukin 8-like 1 [Source:NCBI gene;Acc:395872]
IL6	4.338	0.174	0.157	1.000	0.813	0.234	interleukin 6 [Source:NCBI gene;Acc:395337]
CCL17	3.736	0.002	-0.991	0.373	3.496	0.003	C-C motif chemokine ligand 17 [Source:NCBI gene;Acc:415652]
CCL19	4.879	0.000	2.756	0.001	0.639	0.214	C-C motif chemokine ligand 19 [Source:NCBI gene;Acc:427406]
CCL4	5.436	0.016	1.582	0.191	2.607	0.000	C-C motif chemokine ligand 4 [Source:NCBI gene;Acc:395468]
TLR3	2.236	0.000	1.493	0.019	0.858	0.054	toll like receptor 3 [Source:NCBI gene;Acc:422720]
TLR4	1.232	0.001	0.200	0.711	0.688	0.062	toll like receptor 4 [Source:NCBI gene;Acc:417241]
IRF7	2.089	0.000	0.854	0.140	1.093	0.006	interferon regulatory factor 7 [Source:NCBI gene;Acc:396330]
IRF9	2.433	0.000	0.803	0.197	1.196	0.006	interferon regulatory factor 9 [Source:NCBI gene;Acc:395243]
IFIH1	2.065	0.000	1.146	0.086	0.774	0.211	interferon induced with helicase C domain 1 [Source:NCBI gene;Acc:424185]
IFIT5	3.950	0.000	3.279	0.062	0.357	0.658	interferon induced protein with tetratricopeptide repeats 5 [Source:NCBI gene;Acc:423790]

IFITM5	3.431	0.000	1.104	0.174	1.647	0.001	interferon induced transmembrane protein 5 [Source:NCBI gene;Acc:422992]
IFNW1	Inf	0.136	Inf	1.000	1.709	0.261	interferon omega 1 [Source:NCBI gene;Acc:554219]
IFNG	4.104	0.100	1.775	1.000	0.941	0.614	interferon gamma [Source:NCBI gene;Acc:396054]
MX1	2.834	0.000	1.411	0.080	0.866	0.270	myxovirus (influenza virus) resistance 1, interferon-inducible protein p78 (mouse) [Source:NCBI gene;Acc:395313]
STAT1	2.398	0.001	1.796	0.360	0.527	0.689	signal transducer and activator of transcription 1 [Source:NCBI gene;Acc:424044]
STAT2	3.020	0.000	0.728	0.266	1.652	0.001	signal transducer and activator of transcription 2 [Source:HGNC Symbol;Acc:HGNC:11363]
SOCS1	3.819	0.000	2.098	0.008	1.258	0.005	suppressor of cytokine signaling 1 [Source:NCBI gene;Acc:416630]
SOCS3	2.269	0.000	1.790	0.005	0.274	0.586	suppressor of cytokine signaling 3 [Source:NCBI gene;Acc:395299]
EIF2AK2	2.426	0.000	1.660	0.022	1.045	0.063	eukaryotic translation initiation factor 2 alpha kinase 2 [Source:NCBI gene;Acc:395147]
OASL	3.282	0.000	1.641	0.035	1.222	0.081	2'-5'-oligoadenylate synthetase like [Source:NCBI gene;Acc:395908]
RSAD2	3.013	0.000	3.414	0.187	0.244	0.858	radical S-adenosyl methionine domain containing 2 [Source:NCBI gene;Acc:428650]
